# Milk consumption and the prepubertal somatotropic axis

**DOI:** 10.1186/1475-2891-6-28

**Published:** 2007-09-27

**Authors:** Janet W Rich-Edwards, Davaasambuu Ganmaa, Michael N Pollak, Erika K Nakamoto, Ken Kleinman, Uush Tserendolgor, Walter C Willett, A Lindsay Frazier

**Affiliations:** 1 Connors Center for Women's Health and Gender Biology, Brigham and Women's Hospital, Boston, USA; 2 Department of Ambulatory Care and Prevention, Harvard Medical School and Harvard Pilgrim Health Care, Boston, USA; 3 Department of Epidemiology, Harvard School of Public Health, Boston, USA; 4 Department of Nutrition, Harvard School of Public Health, Boston, USA; 5 Division of Cancer Prevention, Department of Oncology, McGill University, Montreal, Canada; 6 Public Health Institute, Ulaanbaatar, Mongolia; 7 Channing Laboratory, Brigham and Women's Hospital, Harvard Medical School, Boston, USA; 8 Department of Pediatric Oncology, Dana-Farber Cancer Institute, Harvard Medical School, Boston, USA

## Abstract

**Background:**

Nutrients, hormones and growth factors in dairy foods may stimulate growth hormone (GH), insulin-like growth factor I (IGF-I), and raise the ratio of IGF-I to its binding protein, IGFBP-3. We conducted pilot studies in Mongolia and Massachusetts to test the extent to which milk intake raised somatotropic hormone concentrations in prepubertal children.

**Methods:**

In Ulaanbaatar, we compared plasma levels before and after introducing 710 ml daily whole milk for a month among 46 10–11 year old schoolchildren. In a randomized cross-over study in Boston, we compared plasma hormone levels of 28 6–8 year old girls after one week of drinking 710 ml lowfat (2%) milk with their hormone levels after one week of consuming a macronutrient substitute for milk.

**Results:**

After a month of drinking whole milk, Mongolian children had higher mean plasma levels of IGF-I (p < 0.0001), IGF-I/IGFBP-3 (p < 0.0001), and 75^th ^percentile of GH levels (p = 0.005). After a week of drinking lowfat milk, Boston girls had small and non-significant increases in IGF-1, IGF-1/IGFBP-3 and GH.

**Conclusion:**

Milk drinking may cause increases in somatotropic hormone levels of prepubertal girls and boys. The finding that milk intake may raise GH levels is novel, and suggests that nutrients or bioactive factors in milk may stimulate endogenous GH production.

## Background

Milk is a complex, bioactive substance honed by evolution to promote growth and development of the infant mammal. Cow's milk and dairy products derived from milk are widely consumed by human children and adults well after the age of weaning. The established health benefits of the macronutrient and micronutrient content of milk include fewer dental caries, increased bone mineral content, fewer fractures, and reduced risk of protein-deficiency malnutrition and rickets [1-6]. However, the impact of other bioactive substances in milk has been little investigated. Dairy products contain bovine hormones and growth factors that may alter the hormone and growth factor levels of prepubertal children.

Several bovine enzymes, hormones, and growth factors are selectively compartmentalized in milk [7,8]. Although many hormones and growth factors are destroyed or deactivated during digestion and first-pass hepatic metabolism, several lines of evidence indicate that some hormones or growth factors in milk may survive digestion and remain bioactive in the plasma of milk-drinkers.

Rats fed lowfat (1%) cow's milk from the Japanese grocer had higher levels of IGF-I and estrone sulfate than animals fed a macronutrient substitute for milk [9]. Several studies of radioactively labeled IGF-I have demonstrated its intact oral absorption and plasma bioactivity in neonate and adult animals, especially when the IGF-I is administered with the protease inhibitor casein, the primary protein in milk [10-15]. Human studies have consistently shown that high milk consumption is associated with a 10%–20% increase in circulating IGF-I levels among adults and a 20%–30% increase among children [3,16-22]. Although the proteins in milk could be responsible for the higher IGF-I levels in milk-drinkers, dairy products raise IGF-I levels more than do meat and other sources of dietary protein [17-21]. The ratio of IGF-I to its main binding protein, IGFBP-3, also rises with milk consumption, indicating an increase in bioavailable IGF-I [16-18,20]. Two studies have recently reported that milk intake during pregnancy results in higher birthweight offspring [23,24]. The rise in IGF-1 with milk consumption may increase rates of twinning [25].

Most of the steroid hormones and growth factors produced by cows are identical to those produced by humans [26]. If bovine hormones survive the digestive process, most are presumably bioactive. To our knowledge, there has been no macronutrient-controlled experimental investigation of the impact of hormones and growth factors from cow's milk on the plasma hormone and growth factor levels of children. We report here the results of two short-term pilot studies conducted to justify a long-term study of the impact of milk hormones and growth factors on child development.

## Methods

### 1. Mongolian Pilot Study

As children in the US consume large amounts of dairy products, it is difficult to identify a control group with low dairy intake for long-term study. We turned to Mongolia as a possible site for a long-term study. Mongolia has a pastoralist tradition, and milk is a staple of nomadic herders who live on the steppe. Lactose intolerance is virtually unknown. However, a sharp rise in urban migration has resulted in a large group of children whose families lack access to familial herds. Until recently, the only milk available in the capitol city, Ulaanbaatar, was prohibitively expensive imported milk. Ulaanbaatar's children lack access to milk. In Mongolia, we were able to add milk to children's diets, rather than trying to subtract it from the diets of American children. We report here the results of a study conducted to determine the feasibility of a long-term trial of the impact of milk consumption on child development in Ulaanbaatar.

#### Participants

With the approval of the Mongolian Ministry of Education and Ministry of Health Ethical Review Board, we conducted a one-month pilot study in Ulaanbaatar from May to June, 2005. The aim of the study was to determine whether participating in research was acceptable to Mongolian parents, children, and institutions, and to test the logistics of shipping milk and blood samples internationally. A letter was sent to the families of all children in a 3^rd ^grade classroom in Public School #65 in Ulaanbaatar, which included fifty 9–11 year old girls and boys. Families were invited to an informational meeting at the school. The Mongolian investigators (Drs. G and T) explained the study protocol and obtained informed consent from parents and assent from children to participate. Children were eligible if they had no known allergy to milk. One child elected not to enroll, two children responded too late to be enrolled, and one child was withdrawn from the study after developing symptoms of milk allergy. A total of 22 girls and 24 boys completed the protocol.

#### Procedures

We performed a physical examination of each child at the beginning and end of the month, with a Tanner Stage evaluation at the first examination only. At baseline, children completed a food frequency questionnaire regarding their consumption of dairy products in the last seven days. During the intervention month, children consumed three 8-ounce tetrapak "boxes" (710 ml) of conventional U.S. UHT-pasteurized vitamin D fortified whole milk daily. The producer of the milk accepts milk only from dairies that do not use bovine somatotropin (BST). From days 4–10, four children who experienced digestive upset drank varying amounts of milk (236, 472, or 710 ml) as they grew accustomed to the milk. After day 10, all 46 children drank the full 710 ml daily.

#### Hormone comparisons

At the beginning and end of the month, a morning 8 ml blood sample was drawn into a heparinized tube. Blood samples were immediately chilled, and transported cold to a central laboratory, where serum was separated and stored at -70°C. Serum levels of growth hormone, IGF-I, and IGFBP-3 were determined in all samples. We compared pre-intervention and post-intervention hormone levels for each child.

### 2. Boston Girls and Milk Study

#### Participants

We conducted a randomized crossover milk feeding study among prepubertal girls, comparing their hormone and growth factor levels after a week of consuming conventional lowfat (2%) milk compared with a week of consuming a vegetable-based macronutrient substitute for milk. The study was conducted at the Boston Children's Hospital General Clinical Research Center, and was approved by the Children's Hospital Committee on Clinical Investigation. Thirty-one 6–8 year old girls were recruited from several pediatric clinics and by contacting women who had visited the Cancer Risk and Prevention Clinic of the Dana Farber Cancer Institute. Girls were eligible for the study if their height, weight and body mass index were between the 5^th ^and 95^th ^percentile for age, they had no history of endocrine disorders or clinical signs of pubertal initiation, and they had no allergy to milk or the milk substitute. We obtained consent of parents and assent of the girls for a screening physical and taste-test of the milk substitute. A pediatrician (Dr. F) performed a brief physical to ensure that each girl was in Tanner Stage 1, and the girls tested the palatability of the milk substitute before completing a second consent and assent procedure to enroll in the trial. Three girls did not complete the protocol, due to distaste for the milk substitute, fear of blood draw, or inaccessible veins. We present results for 28 girls who completed the trial. Most girls were Caucasian; 2 were African American, 1 was Native American, and 6 were of Asian descent.

#### Procedures

The protocol was five weeks long, including two intervention weeks and an intervening three-week 'wash-out' return to normal diet. In one intervention week, girls consumed 710 ml of 2% conventional cow's milk per day (from a manufacturer that does not use milk from cows treated with BST). In a second intervention week, the girls drank 710 ml daily of a macronutrient substitute for milk with the same calorie, protein, fat, carbohydrate, calcium and vitamin D content as 2% cow's milk, concocted from almond milk, coconut milk, and protein powder. During these two intervention weeks, girls consumed otherwise dairy-free diets. The order of the intervention weeks was randomized. At the introductory visit, parents completed a food frequency questionnaire about their daughter's consumption of dairy products over the past year. Girls consumed 99% of the milk and 92% of the milk substitute.

#### Hormone comparisons

An afternoon 10 ml blood sample was drawn into a heparinized tube at the end of each intervention week, centrifuged, aliquotted, and stored at -70°C. We compared the hormone and growth factor level of each girl at the end of the milk week to her level at the end of the milk substitute week.

### 3. Hormone and growth factor analysis for both studies

GH, IGF-I, and IGFBP-3 were assayed by enzyme-linked immunoabsorbent assay (ELISA) with reagents from Diagnostic Systems Laboratory (Webster, Texas). The IGF-I values obtained by ELISA in our laboratory are highly correlated (Pearson r = 0.97) with values obtained by radioimmunoassay after acid chromatography. The average intra-assay coefficients of variation for IGF-I, IGFBP-3, and GH were 4.9%, 9.0%, and 8.0%, respectively. The IGF-I assay was run in two batches, with limits of detection of 14 ng/ml and 21 ng/ml, respectively (both samples for each child were included in the same batch). The limits of detection were 2 ng/ml for the IGFBP-3 assay and 0.2 ng/ml for the GH assay. Results below the limit of detection were assigned the limit of detection for analysis.

### 4. Statistical analysis for both studies

We performed paired t-tests of differences in the means of IGF-I and the IGF-I/IGFBP3 ratio; the GH distribution was too skewed to apply a parametric test. We used unpaired quantile regression to test differences in the 50^th ^and 75^th ^percentiles of the distributions. We report the 75^th ^percentile because more than half the GH values were at or below the limit of detection, and the 75^th ^percentile captured variation above this "floor" limit. Paired statistical techniques for quantile regression are not available; however, unpaired analyses are valid but would be less powerful than paired techniques if they existed. To display results graphically, we estimated kernel density distributions of hormone and growth factor levels [27]. All analyses were performed using SAS (version 9.1) software.

## Results

Characteristics of children in the two pilot studies are presented in Table 1. Mongolian children are somewhat smaller and leaner than their U.S. peers, with a profile similar to that of U.S. 9–10 year olds [28]. The height, weight and body mass index (BMI, kg/m^2^) of the Boston girls was typical for U.S. girls age 6–8 years [28]. The Mongolian children consumed an average 0.6 servings of whole milk, 0.7 servings of cheese/week, and 1.6 servings of yoghurt in the week before the intervention. Fifteen percent of children had no dairy intake at all, confirming anecdotal reports of low dairy intake among Mongolian urban children. The Boston girls typically consumed 15 glasses of predominantly lowfat or skim milk per week, with an average 19 servings of other dairy products per week.

**Table 1:** Mean (sd) baseline characteristics of participants in two pilot studies

	Mongolian boys	Mongolian girls	Boston girls
n	24	22	28
Age	10.4 (0.7)	10.6 (0.6)	7.6 (0.9)
Weight (kg)	29.1 (0.7)	28.8 (1.0)	25.6 (4.6)
Height (cm)	132.5 (1.4)	130.8 (1.3)	123.9 (6.6)
BMI (kg/m^2^)	16.5 (0.3)	16.8 (0.3)	16.6 (2.0)
Tanner stage >1	12% (3/24)	4% (1/22)	0% (by design)
Per week:			
Servings of milk	0.8 (1.3)	0.5 (1.0)	14.9 (5.8)
Servings of other dairy^1^	6.5 (6.0)	4.1 (4.0)	18.9 (10.3)

1. "Other dairy" for the Boston girls included cheese, ice cream, yoghurt, and cheese-containing dishes such as pizza or pasta. "Other dairy" for the Mongolia children consisted of cheese, yoghurt, or milk added to tea.

**Figure 1:**
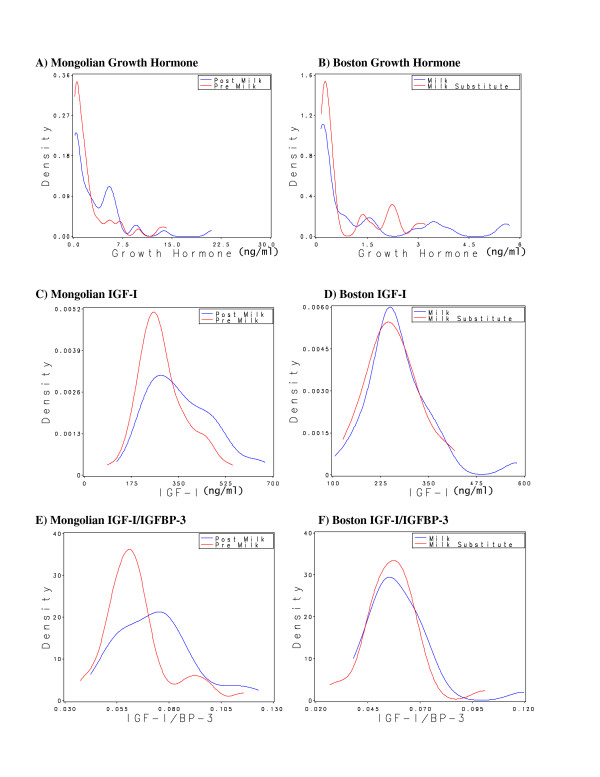
**Distributions of plasma somatotropic hormones in two pilot studies**. The Mongolia study compared concentrations of growth hormone (GH) (1a), insulin-like growth factor I (IGF-I) (1b), and the ratio of IGF-I to its binding protein 3 (IGF-I/IGFBP-3) (1c) among 9–11 year old Mongolian boys and girls (n = 46) before (red line) and after (blue line) one month of drinking 710 ml whole milk daily. The Boston study included 6–8 year old girls (n = 28) whose concentrations of GH (1d), IGF-I (1e), and the ratio of IGF-I/IGFBP-3 (1f) were measured after 1 week consuming 710 ml of lowfat milk daily (blue line) and one week of consuming a vegetable-based macronutrient substitute for milk (red line). **a) **Mongolian GH. **b) **Boston GH. **c) **Mongolian IGF-I. **d) **Boston IGF-I. **e) **Mongolian IGF-I/IGFBP-3. **f) **Boston IGF-I/IGFBP-3.

Figure 1 shows the kernel density estimated distributions of GH, IGF-I, and IGF-I/IFGBP-3 according to milk intervention arm in each study. As expected, the distribution of GH in both studies was skewed, with the median values in both studies at or near the limit of detection. The highest GH values were observed after milk consumption in both studies. In Mongolia, large increases in IGF-I and IGF-I/IGFBP-3 levels were observed between the beginning and end of the milk intervention month. In Boston, there was a subtle shift of the IGF-I and IGF-I/IGFBP-3 distributions to higher levels after a week of consuming milk, compared with a week of consuming milk substitute.

During the milk intervention month in Mongolia, girls grew a mean 1.1 (0.2 sd) centimeters, 0.65 (0.2) kg, and had a mean drop in BMI of -0.29 (0.06). Boys grew a mean 1.0 (0.2) centimeters, 0.23 (0.1) kg, and had a mean decrease in BMI of -0.26 (0.06). Consistent with the rapid increase in height over the intervention month, we observed large increases in serum GH, IGF-I, and IGF-I/IGFBP-3 (Table 2). Large differences in the means, medians, and 75^th ^percentile of these somatotropic hormones were statistically significant, with the exception of median GH, which was nearly doubled but was not statistically significant. Changes in GH and growth factor levels from the beginning to the end of the milk intervention month were similar for girls and boys (data not shown).

**Table 2:** Change in 10 year-old Mongolian children's hormone and growth factor concentrations before and after one month of drinking 710 ml of American UHT whole milk daily.

**Mongolia**		**Mean**	**50^th ^percentile **	**75^th ^percentile**
**Growth Hormone (ng/ml)**	Pre	2.38 ± 3.27	1.23	2.60
	Post	3.42 ± 4.07	2.14	5.28
	p-value	NA*	0.23	0.005

**IGF-I (ng/ml)**	Pre	290.93 ± 93.98	270.70	346.95
	Post	358.34 ± 125.62	333.56	449.29
	p-value	<0.0001	0.04	0.03

**IGF-I/IGFBP-3**	Pre	0.064 ± 0.016	0.062	0.068
	Post	0.073 ± 0.019	0.072	0.082
	p-value	<0.0001	0.009	0.007

For means, p-values are from paired t-tests. For 50^th ^and 75^th ^percentiles, p-values are from unpaired quantile regression.

* T-test comparing mean GH not appropriate, due to non-Normal GH distribution.

Table 3 shows the results of statistical tests of the plasma assays in the Boston trial. The mean, median, and 75th percentiles were higher during the week in which the girls drank milk, but these differences were neither large nor statistically significant in these 6–8 year olds.

**Table 3:** Comparison of hormone and growth factor levels among 6–8 year old Boston girls after 1 week milk consumption versus 1 week macronutrient substitute for milk (n = 28).

**Boston**		**Mean**	**50^th ^percentile **	**75^th ^percentile**
**Growth Hormone (ng/ml)**	After substitute	0.89 ± 0.96	0.41	1.52
	After milk	1.25 ± 1.67	0.21	1.62
	p-value	NA*	0.63	0.93

**IGF-I (ng/ml)**	After substitute	257.26 ± 69.12	250.36	294.71
	After milk	270.60 ± 89.46	250.20	308.77
	p-value	0.35	0.99	0.68

**IGF-I/IGFBP-3**	After substitute	0.057 ± 0.01	0.057	0.064
	After milk	0.059 ± 0.02	0.056	0.068
	p-value	0.28	0.72	0.28

For means, p-values are from paired t-tests. For 50^th ^and 75^th ^percentiles, p-values are from unpaired quantile regression.

*T-test comparing mean GH is not appropriate, due to non-Normal GH distribution

## Discussion

In a Mongolian pilot study, levels of prepubertal GH, IGF-I, and IGF-I/IGFBP-3 were markedly higher after one month of drinking milk. In the smaller, shorter Boston pilot study, which contrasted cow's milk to a macronutrient control for cow's milk, levels of GH, IGF-I and IGF-I/IGFBP3 were not elevated, although we observed a small non-significant shift in somatotropin levels in the direction of the Mongolian findings. The rise in somatotropic hormone and growth factor levels was larger in the Mongolian than in the Boston study, perhaps due to the longer intervention, the fact that the Mongolian children consumed less dairy at baseline, the use of whole versus lowfat milk, the older age of the Mongolian participants, or the baseline nutritional status of the Mongolian children.

We conducted the Mongolian feasibility pilot with limited resources, and therefore did not attempt a macronutrient control. During the intervention month, the Mongolian children experienced rapid linear growth (the equivalent of 12 cm/year) compared to an average height velocity of 5–6 cm/year in U.S. children age 10–11 years [29]. Although such rapid growth suggests that the children may have been undernourished at the start of the trial, the increase in GH is unlikely to be a refeeding effect of milk nutrients. The classic hormonal profile in undernutrition is low IGF-I, but stable or even elevated GH levels [30-33]. High GH during fasting may promote the tissue-sparing switch from gluconeogenesis to lipolysis during periods of undernutrition [30,31]. On refeeding, IGF-I levels increase, but GH levels typically fall [31,32]. In the Mongolian children, the increase in GH levels after milk consumption was proportionally higher than the increase in either IGF-I or IGF-I/IGFBP-3 levels. This finding is inconsistent with a malnutrition correction effect. As GH is released in a pulsatile fashion, and we had only one GH measurement in these pilot studies, we undoubtedly overestimated the variability of GH, reducing our statistical power to detect a GH response to milk. Future studies should measure GH more frequently to capture GH secretory dynamics and better estimate GH levels.

Our studies suggest an immediate stimulatory impact of milk on the secretion of GH and bioactive IGF-I. Other studies suggest that the impact of milk on IGF-I levels is maintained, and may grow over time. In China, 10 year old girls randomized to consume whole milk had 9% higher IGF-I levels after one year, and 20% higher levels after two years (p < 0.05) [2]. In Britain, 12 year-old girls randomized to drink approximately 300 ml/day of milk had 10% higher IGF-I levels after 6 months of intervention, and 17% higher levels at months 12 and 18 of intervention (p = 0.02) [3]. To our knowledge, ours is the first study to report GH response to dairy intake, which was large and statistically significant among the Mongolian children.

The findings here should be replicated in larger studies with macronutrient and micronutrient control. If consistent findings emerge, it would be important to identify the specific bioactive factor in bovine milk that raises GH and IGF-I levels. Although human and bovine GH are not identical, bovine GH absorbed from milk might cross-react with the human GH assay, and it is conceivable (but as yet untested) that bovine GH might stimulate human IGF-I production. Animal feeding experiments, using radioactively labeled bovine vs. human GH, would be an approach to answering the question of the origin of elevated GH we observed in the children's serum. Alternatively, another factor in bovine milk may stimulate endogenous production of human GH, which would, in turn, provoke IGF-I production. Although bovine and human IGF-I are identical, IGF-I absorbed from milk is unlikely to stimulate human GH secretion, as GH levels should drop in response to negative feedback from IGF-I infusion. Future analysis should focus on identifying factors in milk that could stimulate human GH secretion, such as growth hormone releasing hormone, ghrelin, or a substance that stimulates ghrelin release.

## Conclusion

Milk drinking may cause increases in somatotropic hormone levels of prepubertal girls and boys. The finding that milk intake may raise GH levels is novel, and suggests that nutrients or bioactive factors in milk may stimulate endogenous GH production.

## Abbreviations

BMI = body mass index (kg/m^2^)

BST = bovine somatotropin

GH = growth hormone

IGF-I = insulin-like growth factor I

IGFBP-3 = insulin-like growth factor binding protein 3

## Competing interests

The author(s) declare that they have no competing interests.

## Authors' contributions

JRE, DG, LF, WW, and EN designed the study. DG and UT conducted the Mongolian pilot study. LF, JRE, and EN conducted the Boston study. MP contributed hormone assays and interpretation. KK and EN performed statistical analyses. JRE drafted the manuscript, and all contributors have edited and approved the manuscript.
